# International Consensus Classification of myeloid and lymphoid neoplasms: myeloproliferative neoplasms

**DOI:** 10.1007/s00428-022-03480-8

**Published:** 2022-12-29

**Authors:** Umberto Gianelli, Jürgen Thiele, Attilio Orazi, Naseema Gangat, Alessandro M. Vannucchi, Ayalew Tefferi, Hans Michael Kvasnicka

**Affiliations:** 1 University of Milan, Department of Health Sciences and S.C. Anatomia Patologica, ASST Santi Paolo e Carlo, Milan, Italy; 2 Institute of Pathology, University of Cologne, Cologne, Germany; 3 Department of Pathology, Texas Tech University Health Sciences Center, El Paso, TX USA; 4 Mayo Clinic, Rochester, MN USA; 5 CRIMM-Centro Ricerca e Innovazione delle Malattie Mieloproliferative, Azienda Ospedaliera-Universitaria Careggi, Department of Experimental and Clinical Medicine, University of Florence, Florence, Italy; 6 University Clinic Wuppertal, University of Witten/Herdecke, Wuppertal, Germany

**Keywords:** International Consensus Classification, Myeloid and lymphoid neoplasms: Myeloproliferative neoplasms

## Abstract

The recently published International Consensus Classification (ICC) of myeloid neoplasms summarized the results of an in-depth effort by pathologists, oncologists, and geneticists aimed to update the 2017 World Health Organization classification system for hematopoietic tumors. Along these lines, several important modifications were implemented in the classification of myeloproliferative neoplasms (MPNs). For chronic myeloid leukemia, *BCR*::*ABL1-*positive, the definition of accelerated and blast phase was simplified, and in the *BCR*::*ABL1-*negative MPNs, the classification was slightly updated to improve diagnostic specificity with a more detailed and better validated morphologic approach and the recommendation of more sensitive molecular techniques to capture in particular early stage diseases. In this regard, high sensitive single target (RT-qPCR, ddPCR) or multi-target next-generation sequencing assays with a minimal sensitivity of VAF 1% are now important for a proper diagnostic identification of MPN cases with low allelic frequencies at initial presentation. This review discusses the updated diagnostic criteria of MPN according to the ICC, particularly by highlighting the new concepts and how they can be applied in clinical settings to obtain an appropriate prognostic relevant diagnosis.

## Introduction

The International Consensus Classification (ICC) of myeloid and lymphoid neoplasms [1] represents the results of an in-depth discussion by hematopathologists from the European Association of Haematopathology (EAHP) and Society for Hematopathology (SH) during the 20th Meeting of the European Association of Haematopathology (Virtual, April 2021) and the collaborative work of hematopathologists, hematologists, and molecular biologists during the Clinical Advisory Committee held in Chicago (September 2021) to update the current classification of myeloid neoplasms [2].

According to the ICC guidelines, the category of myeloproliferative neoplasms (MPN) include *BCR*::*ABL1*-positive chronic myeloid leukemia (CML), essential thrombocythemia (ET), primary myelofibrosis (PMF), and polycythemia vera (PV) as well as chronic neutrophilic leukemia (CNL) and chronic eosinophilic leukemia (CEL).

In the CML group, the main effort of the ICC resulted in a simplified definition of accelerated and blast phase (AP and BP), while in the other MPN subtypes, reduction of diagnostic uncertainty, especially in initial disease stages, and the identification of specific molecular lesions were in focus to optimize clinical management of patients. In all MPN subtypes, high sensitive single target (RT-qPCR, ddPCR) or multi-target panel/next-generation sequencing (NGS) assays with a minimal sensitivity of 1% are recommended to identify molecular alteration even with low variant allelic frequencies (VAF) at initial diagnosis.

This review aims to discuss diagnostic criteria of MPN according to the ICC, particularly by highlighting and elaborating new aspects and their application for diagnosis.

## Chronic myeloid leukemia, *BCR::ABL1-*positive

### Clinical features

Incidence of chronic myeloid leukemia, *BCR::ABL1*–positive, (CML) in the general population accounts for 1–2 cases per 100.00 adults and about 15% of newly diagnosed cases of leukemia in adults [3]. Since the introduction of tyrosine kinase inhibitors (TKI) in 2000 annual mortality of CML has decreased from 10–20% to 1–2%, with significant increase of the prevalence in well-developed countries and an improvement of the 10-year survival rate from approximately 20% to 80–90% [4]. However, in cases with ineffective therapy, CML may evolve into AP and BP (myeloid in about 60% of the cases, lymphoid in 30%, and megakaryocytic or undifferentiated in 10%). Importantly, leukemic evolution can present also without a previous AP. Diagnosis of chronic phase CML (CP-CML) which is mainly based on the detection of the *BCR::ABL1* rearrangement remained unchanged, while the diagnostic criteria for AP and BP have been simplified by the ICC CML working group. The ICC guidelines have maintained a blast percentage threshold of 10–19% and at least 20% in the blood or BM to establish the diagnosis of AP and BP, respectively. Of note, other classification systems which include the International Blood and Marrow Transplant Registry (IBMTR), M.D. Anderson Cancer Center (MDACC), and the European LeukemiaNet have defined a higher blast threshold of more than 30% and are more frequently used as eligibility criteria in clinical trials. According to the ICC criteria, AP is defined by 10–19% bone marrow or peripheral blood blasts, peripheral blood basophilia > 20%, or the identification of additional clonal cytogenetic abnormalities. Previous criteria for AP that have been included in the WHO 2017 definition like thrombocytopenia (≤ 100  ×  10^9^/L) unrelated to therapy or unresponsive thrombocytosis (> 1000  ×  10^9^/L) and/or splenomegaly to therapy have been discarded by the ICC CML working group. Furthermore, the failure to achieve complete hematological response or resistance to sequential tyrosine kinase inhibitors, or occurrence of > 2 mutations on *BCR::ABL* during treatment, has also been eliminated in the definition of AP. CML-BP is characterized by 20% or more myeloid blasts or extramedullary myeloid sarcoma. Importantly, the presence of > 5% lymphoid blasts in peripheral blood or bone marrow is defining lymphoblastic crisis and thus should prompt further laboratory and genetic studies [5–7]. In established AP or BP, or in patients with clinical features suggesting disease progression (e.g., progressive splenomegaly), bone marrow evaluation is recommended. Noteworthy, mild increase in bone marrow fibrosis (MF-1), even at initial diagnosis, correlates with a decreased major molecular response (MMR) rate in the first year of TKI therapy [8, 9].

### Morphology

In CP-CML, peripheral blood displays leukocytosis (median value: 80 × 10^9^/L) with neutrophils in various stages of maturation and increase of myelocytes and segmented neutrophils without significant dysplasia [6].﻿ Absolute basophilia and eosinophilia are frequent findings. It should be noted that some patients lack significant leukocytosis and present with a sustained thrombocytosis mimicking ET at initial diagnosis. However, the majority of patients is diagnosed in the setting of a persistent unexplained leukocytosis, and the diagnosis of CP-CML is established by the characteristic Philadelphia (Ph) chromosome abnormality t(9; 22)(q34;q11), assessed either by routine cytogenetics or the detection of a *BCR::ABL1* abnormality by fluorescence in situ hybridization or molecular studies. Although a bone marrow biopsy is not required at diagnosis, baseline evaluation of the hematopoietic series and of the grade of bone marrow fibrosis by reticulin staining can be prognostically informative. In CP, the bone marrow is hypercellular for the patient’s age with marked granulocytic proliferation with a left-shift like in the peripheral blood, decreased erythroid precursors, and an increased number of small megakaryocytes (in about 40–50% of the cases) with hypolobulated nuclei (“dwarf” megakaryocytes). Blasts usually account for less than 5% [10, 11]. Eosinophils and basophils are usually increased in number, and pseudo-Gaucher histocytes may be observed. Of note, cases carrying the p230 fusion protein often show a marked neutrophilic maturation and thrombocytosis, while those cases associated with a p190 fusion protein may present with an increased number of mature monocytes mimicking chronic myelomonocytic leukemia. In AP, the increased blast count can be associated with dysplastic changes in the granulocytic precursors and megakaryocytes (i.e., myelodysplasia-like micro-megakaryocytes) together with an accumulation of reticulin/collagen fibers.

### Genetic profile

In 95% of CML cases, the characteristic t(9;22)(q34.1;q11.2) translocation defined as Philadelphia chromosome is present. This translocation is responsible for the fusion gene *BCR::ABL1* and the consequently chimeric protein p210. Different breakpoints and rearrangements can result in about 1% of patients in a shorter p190 oncoprotein and in 2–5% of patients in a p210 variant or p230 transcript which usually is associated with a more indolent behavior [12]. Most of the patients show only the Ph chromosome throughout the chronic phase. During CML progression to AP and BP, secondary chromosomal abnormalities can be detected, most commonly +8 (34% of cases with additional changes), +Ph (30%), i(17q) (20%), +19 (13%), -Y (8% of males), +21 (7%), +17 (5%), and monosomy 7 (5%), which are often associated with an unfavorable prognosis [13]. The acquisition of major-route additional chromosomal abnormalities on treatment is considered as hallmark of disease progression. About 30% of CML patients in CP with resistance to first or second-generation of tyrosine kinase inhibitors (TKIs) harbor mutations in the *BCR::ABL1* kinase domain. Additional mutations indicate a risk disease with higher rate of relapse on second- or subsequent line of therapy and further acquisition of molecular abnormalities. Consequently, identification of *BCR::ABL1* mutations in patients treated with TKIs represents a biological hallmark of disease progression. In this context, NGS is highly sensitive to identify emerging resistant mutations even at the time of major or deeper molecular responses [14].

Diagnostic criteria for AP and BP CML are reported in Table 1.

**Table 1:** Diagnostic criteria for accelerated and blast phase chronic myeloid, BCR::ABL1–positive (CML) according to the International Consensus Classification^1^

Accelerated phase	Bone marrow or peripheral blood blasts 10–19%	Blast phase	Bone marrow or peripheral blood myeloid blasts ≥ 20%
	Peripheral blood basophils ≥ 20%		Myeloid sarcoma^b^
	Presence of additional clonal cytogenetic abnormality in Ph+ cells (ACA)^a^		Bone marrow or peripheral blood lymphoid blasts > 5% is consistent with lymphoblastic crisis^c^

*Ph*, Philadelphia chromosome

^a^Second Ph, trisomy 8, isochromosome 17q, trisomy 19, complex karyotype ≥ 3 cytogenetic abnormalities, or abnormalities of 3q26.2

^b^Extramedullary blast proliferation

^c^Immunophenotypic analysis is required to confirm lymphoid lineage

## Essential thrombocythemia

### Clinical features

ET incidence is estimated at 1.2 to 3.0 per 100,000 population per year [15] with a median age at diagnosis of 58 years and a slight female predominance. More than 50% of the patients are asymptomatic and discovered incidentally with thrombocytosis (by definition > 450 × 10^9^/L). Symptoms are more frequently associated with thrombosis (ranging from transient ischemic attacks involving small vessels to splanchnic vein thrombosis) or hemorrhages (more frequently involving the gastrointestinal and respiratory tracts) [16, 17]. Mild splenomegaly can be seen in about 15–20% of the cases, while hepatomegaly is rare. Thrombohemorrhagic complications represent two of the main causes of morbidity and mortality in these patients [18, 19]. In a large cohort of patients, the rate of fatal and non-fatal thrombotic events was 1.9% per patient/year [17]. Progression to post-ET MF has a cumulative risk at 10 years ranging between 0.8 and 4.5%. Similar to CML, in *BCR::ABL1*-negative MPN, progression to AP is defined by the presence of 10 to 19% of peripheral blood or bone marrow blasts, while BP is defined by the presence of 20% or more blasts. Progression to AP at 10 years has been reported to range between 0.7 and 3% [20] and by strict adherence to the previous WHO criteria only between 0.7 and 1.9 % [21–23]. Moreover, advanced age, extreme thrombocytosis, anemia, leukocytosis, and additional mutations involving *TP53* and *EZH2* have been reported as risk factors for BP progression [24, 25]. Median overall survival in ET patients ranges from 14.7 to about 21.8 years [21, 26].

### Morphology

**Fig. 1:**
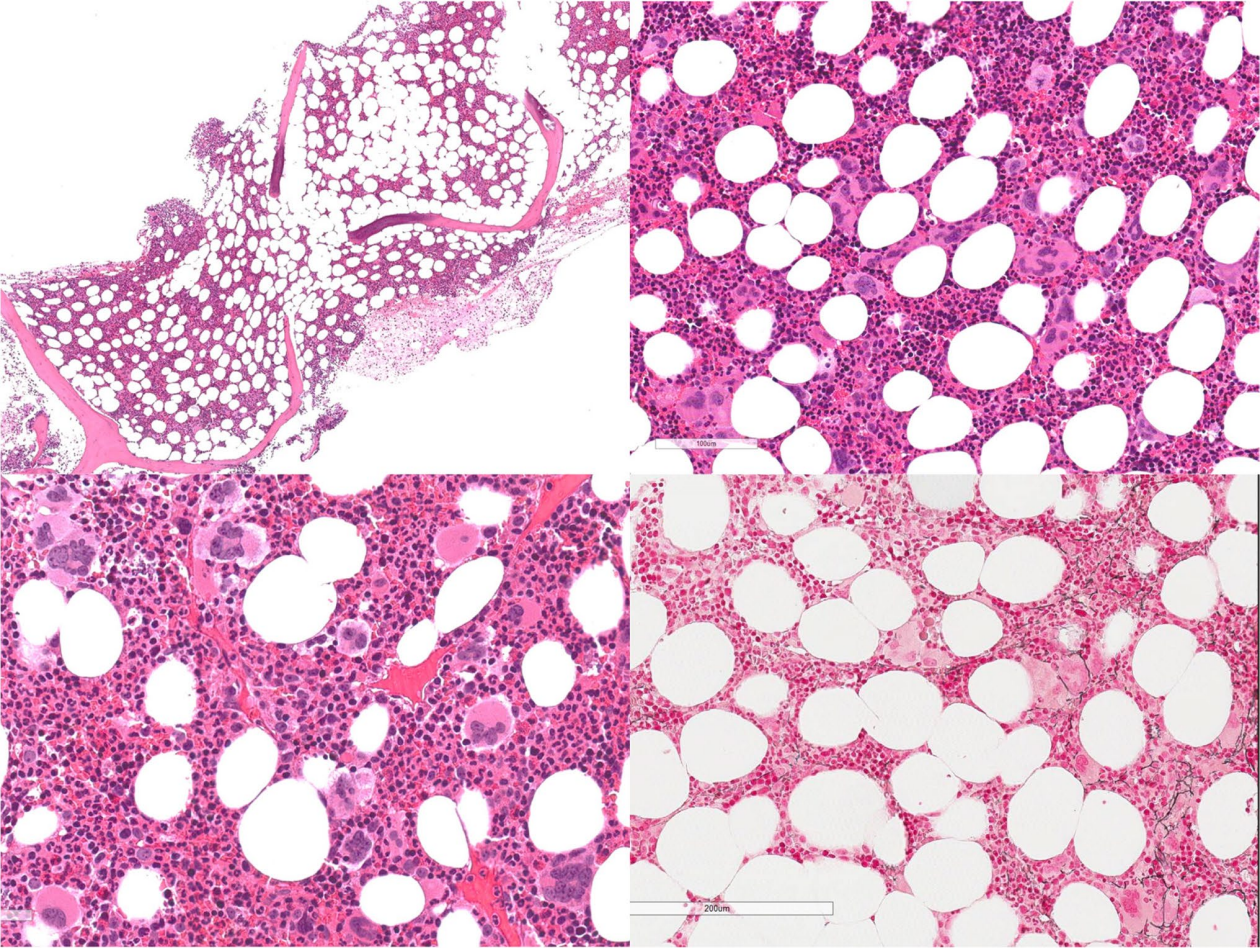
Essential Thrombocythemia is characterized by a normocellular marrow with increased number of megakaryocytes forming loose clusters and rarely dense clusters, with large to giant elements and hyperlobulated nuclei. Erythropoietic and granulopoietic series do not show significant abnormalities. Reticulin fibrosis is usually not increased (MF-0)

In the peripheral blood, the most frequent anomaly consists of thrombocytosis usually associated with anisocytosis of platelets. Bone marrow is normocellular for the patients’ age, with only a few cases showing a mild hypercellularity (Fig. 1). Erythropoiesis, granulopoiesis, and myeloid/erythroid ratio do not show significant abnormalities. Megakaryocytes are increased in number, usually large to giant with hyper-lobulated nuclei and abundant mature cytoplasm. Frequently loose clusters can be observed, and only very rarely they aggregate in dense clusters (usually small clusters with less than 6 cells). In these cases, the differential diagnosis with pre-fibrotic PMF might be challenging; however, identification of atypical megakaryocytes, presence of granulocytic proliferation, and clinical features like increased LDH or splenomegaly support the diagnosis of pre-fibrotic PMF. In detail, megakaryocytic atypia in PMF consists of nuclear and cytoplasmic abnormalities (increased nuclear/cytoplasmic ratio, irregular chromatin clumping, bulbous appearance, marked hyperchromasia). Myeloblasts are usually less than 5%, and a mild increase in reticulin fibers (grade 1) can be observed in less than 5% of patients at initial diagnosis [27–29].

### Genetic profile

A *JAK2V617F* driver mutation can be found in about 60% of ET cases, *calreticulin* (*CALR*), and *MPL* mutations in about 20% and 3%, while only a small subgroup of patients presents without one of these driver mutations (so-called “wild-type”) [21, 30]. *JAK2V617F* has been associated with an increased risk of thrombosis and a lower risk of myelofibrotic progression, i.e., post-ET MF [22]. On the other side, *CALR*-positive patients are younger, more frequently male, and characterized by higher platelet counts, lower hemoglobin level, lower leukocyte count, and lower incidence of thrombotic events. In this context, type 2 vs type 1 *CALR* mutations were associated with higher platelet count [31, 32]. NGS analysis revealed that 53% of ET cases harbor one or more additional variants, other than *JAK2* V617F/*CALR/MPL.* The most frequent were *TET2* and *ASXL1* [33]. Adverse variants for decreased overall, leukemia- or fibrosis-free survival included *SH2B3*, *SF3B1*, *U2AF1*, *TP53*, *IDH2*, and *EZH2*. Overall survival is impacted by *SF3B1/SRSF2* mutations, whereas *U2AF1* and *SF3B1* mutations may affect myelofibrosis-free survival and TP53 mutations predicted leukemic transformation. In this regard, assessment of MPN drivers and high molecular risk (HMR) mutations allow the calculation of a mutation-enhanced international prognostic system [34].

Diagnostic criteria for the diagnosis of ET according to the ICC are reported in Table 2.

**Table 2:** Diagnostic criteria for essential thrombocythemia and post-essential thrombocythemia myelofibrosis (post-ET MF) according to the International Consensus Classification^1^

	ET		Post-ET MF
Major criteria	1. Platelet count ≥ 450 × 10^9^/L	Required criteria	1. Previous established diagnosis of ET
	2. Bone marrow biopsy showing proliferation mainly of the megakaryocytic lineage, with increased numbers of enlarged, mature megakaryocytes with hyperlobulated staghorn-like nuclei, infrequently dense clusters^a^; no significant increase or left shift in neutrophil granulopoiesis or erythropoiesis; no relevant BM fibrosis^b^		2. Bone marrow fibrosis of grade 2 or 3 (MF-2 or MF-3)
	3. Diagnostic criteria for *BCR::ABL1* positive chronic myeloid leukemia, polycythemia vera, primary myelofibrosis, or other myeloid neoplasms are not met	Additional criteria	Anemia (i.e., below the reference range given age, sex, and altitude considerations) and a > 2 g/dL decrease from baseline hemoglobin concentration
	4. *JAK2*, *CALR*, or *MPL* mutation^c^		Leukoerythroblastosis
Minor criteria	1. Presence of a clonal marker^d^ or absence of evidence of reactive thrombocytosis^e^		Increase in palpable splenomegaly of > 5 cm from baseline or the development of a newly palpable splenomegaly
			Elevated lactate dehydrogenase level above the reference range
			Development of any 2 (or all 3) of the following constitutional symptoms: >10% weight loss in 6 months, night sweats, unexplained fever (> 37.5 °C)

The diagnosis of ET requires either all major criteria or the first 3 major criteria plus the minor criteria. The diagnosis of post-ET MF is established by the two required criteria and at least two additional criteria

^a^Three or more megakaryocytes lying adjacent without other BM cells in between; in most of these rare clusters < 6 megakaryocytes may be observed, increase in huge clusters (> 6 cells) accompanied by granulocytic proliferation is a morphological hallmark of pre-PMF

^b^Very rarely a minor increase in reticulin fibers may occur at initial diagnosis (MF-1)

^c^It is recommended to use highly sensitive assays for *JAK2*V617F (sensitivity level < 1%) and *CALR* and *MPL* (sensitivity level 1–3%)—in negative cases, consider a search for non-canonical *JAK2* and *MPL* mutations

^d^Assessed by cytogenetics or sensitive NGS techniques

^e^Reactive causes of thrombocytosis include a variety of underlying conditions like iron deficiency, chronic infection, chronic inflammatory disease, medication, neoplasia, or history of splenectomy

## Primary myelofibrosis

The ICC guidelines aim to increase diagnostic specificity, especially in initial/early cases of MPN presenting with thrombocytosis. Over the last years, several studies clearly confirmed clinical, morphological, and molecular differences between the prefibrotic stage of PMF and ET, and therefore, the definition of a prefibrotic stage as distinct disease category within the MPN subtypes has been maintained [26, 35–37].

### Early/pre-fibrotic primary myelofibrosis

#### Clinical features

According to results from reclassification studies of BM biopsies and corresponding clinical data to differentiate “true” ET from pre-PMF, after centralized evaluations by centers of excellence, the incidence of pre-PMF in cases originally diagnosed as ET may be calculated between 14 and 18% [21, 38, 39]. Approximately, 30–40% of pre-PMF patients are asymptomatic at diagnosis, but reveal an abnormal CBC, usually slight anemia, or leukocytosis and less commonly gross splenomegaly. Thrombocytosis clinically mimicking ET is one of the most common and challenging presentation in pre-PMF. Rarely, unexplained leukoerythroblastosis or an increased lactate dehydrogenase (LDH) level prompts the initial diagnosis. Compared to overt PMF, patients with pre-PMF are often younger and present with higher hemoglobin and platelet counts and minimal leukocytosis [35]. Symptomatic cases reveal constitutional symptoms like fatigue, weight loss, night sweats, and dyspnea. Borderline to minimal splenomegaly represents a common finding (90% of the cases), while hepatomegaly of various degree can be documented in about half of the patients. The median survival in pre-PMF has been reported to range between 11 and 17 years, contrasting only 7 years for overt PMF. Reticulin fibrosis (MF-1) and anemia at initial diagnosis were identified as risk factors for progression from pre-PMF to overt disease stage. Furthermore, variables associated with BP evolution are age > 65 years, leukocytosis (> 15 × 10^9^/L), and LDH ratio > 1.5 times the normal institutional value and cytogenetic abnormalities [40].

#### Morphology

**Fig. 2:**
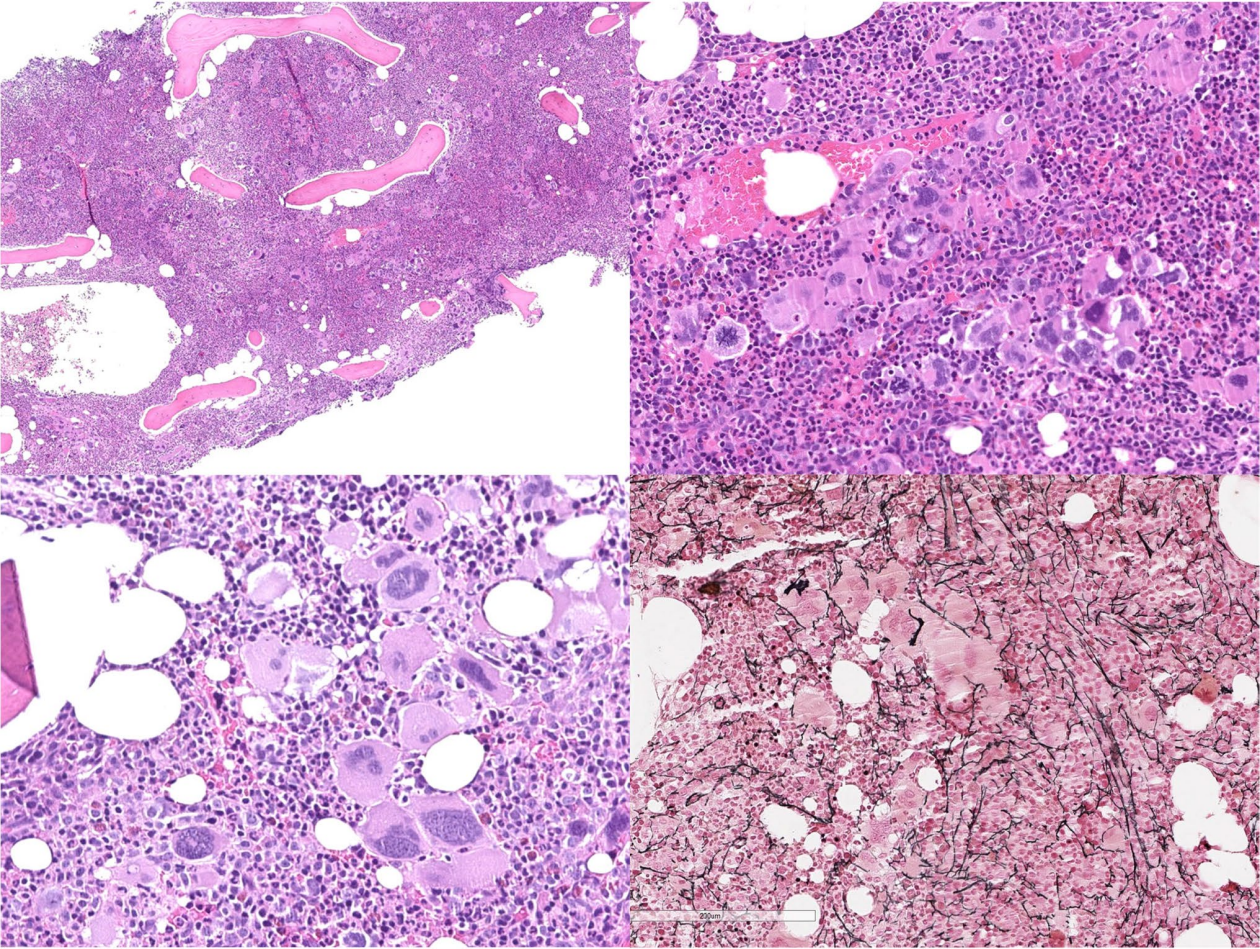
Early/pre-fibrotic PMF is characterized by a hypercellular marrow with pronounced granulopoiesis and increased number of megakaryocytes, usually forming dense clusters and displaying atypical morphology. Erythropoiesis is often reduced, particularly in cases with increased reticulin fibrosis (MF-1)

In pre-PMF, peripheral blood shows a mild anisopoikilocytosis without leukoerythroblastosis. Bone marrow is characteristically hypercellular for the patient’s age with pronounced proliferation and left shifting of granulopoietic precursors and increased myeloid/erythroid ratio (Fig. 2). Megakaryocytes are increased in number and characterized by polymorphisms (variation in size and shape) and atypia (increased nucleus/cytoplasmic ratio, abnormal chromatin clumping, bulbous, and hyperchromatic nuclei) and form abnormal large dense clusters as morphological key feature (major criterion). These huge clusters are defined by more than 6 megakaryocytes lying strictly adjacent without other bone marrow cells in between. It is important to underline that the presence of this abnormal morphological feature is a morphological hallmark of pre-PMF and in general not seen in other MPN subtypes, particularly ET. Therefore, in cases clinically assigned as ET occurrence of large dense clusters (according to the ICC definition) should always prompt a critical reevaluation of diagnosis by inclusion of other important features like increased LDH level, leukocytosis ≥ 11 × 10^9^/L, anemia not attributed to a comorbid condition, and palpable splenomegaly (minor criteria). By definition, reticulin fibrosis is absent (MF-0) or mild (MF-1) in pre-PMF. In some cases of ET, smaller dense clusters of megakaryocytes (< 6 cells) can be found and thus may be a source of diagnostic confusion. In these challenging cases, separation from pre-PMF should be based on the critical evaluation of the complete histological pattern (including immunohistochemistry for megakaryocytes) based on overall cellularity, myeloid/erythroid ratio, and morphology and histotopography of the megakaryocytes (i.e., dense clusters) and stromal changes (i.e., bone marrow fibrosis, osteosclerosis) along with clinical data.

#### Genetic profile

In pre-PMF, abnormal cytogenetics is found in about 18% and unfavorable karyotypes in 4–8% of cases. Unfavorable abnormalities consist in complex karyotype (> 3 abnormalities), isolated +8, isolated −7/7q−, sole abnormalities like i(17q), −5/5q−, 12p−, 11q23 rearrangement or inv(3), and an abnormal karyotype with abnormalities of chromosomes 5, 7, 17, or 12p−. Incidence of *JAK2*V617F mutations is very similar in pre-PMF and ET, ranging between 52–67% and 54–66%, respectively [26, 35]. There is no difference in the distribution of MPN driver mutations (*JAK2*V617F, *MPL*W515x, and *CALR*) between pre-PMF and overt-PMF. *JAK2*V617F mutation was found in 67.2% of pre-PMF and 58.2% of overt PMF, *CALR* type 1 and type 2 in 12.2% and 5.8%, and 17.8% and 4.4% of pre-PMF and overt PMF, respectively; *MPL*W515x-mutated patients were 4.7% and 6.0% in the 2 cohorts. On the contrary, the high mutation risk (HMR) category (any mutations in *ASXL1*, *SRSF2*, *IDH1*, *IDH2*, *EZH2*) is more frequently observed in overt PMF [35]. The proportion of patients lacking any driver mutation (“triple negative PMF”) is similar between pre-PMF and overt PMF ranging between 10.1 and 13.6%. These triple-negative cases belong to a subgroup with high risk of leukemic transformation and very poor prognosis. Most of these triple-negative cases present with thrombocytopenia and only rarely with splenomegaly. A pronounced proliferation of the granulopoiesis as seen in pre-PMF is less likely and dysplastic changes of the erythropoietic elements may be observed. Furthermore, cytogenetics frequently reveals a trisomy 8, and molecular analysis shows an enrichment in high-risk mutations, which overall might trigger the detrimental effect on prognosis. Due to their clinical, morphological, and molecular overlap with the heterogenous group of myelodysplastic/myeloproliferative neoplasms (MDS/MPN), these cases can pose a diagnostic challenge.

### Overt primary myelofibrosis

#### Clinical features

Incidence of overt PMF accounts for about 0.5–1.5 patients × 100.00 population per year. In overt disease, clinical manifestations more frequently include anemia, marked hepatosplenomegaly, constitutional symptoms (e.g., fatigue, night sweats, fever), cachexia, pruritus, and thrombo-hemorrhagic complications. The cumulative incidences of myeloid BP are reported as 11% at 5 years and 23% at 10 years. Causes of death include leukemic progression that occurs in approximately 20% of patients, but many patients also die of comorbid conditions including cardiovascular events and consequences of cytopenia, including infection or bleeding.

#### Morphology

Due to the deposition of reticulin and collagen fibers in the overt stage, overall cellularity progressively decreases including a significant reduction of the erythroid compartment. In the end stages of disease, the intertrabecular marrow spaces can be occupied mainly by collagen fibers, with scattered myeloid precursors and abnormal megakaryocytes which tend to be smaller and more dysmorphic than in early disease stage. Increased micro-vessel density, with dilated and distorted sinusoids, intra-sinusoidal hematopoiesis, and osteosclerosis is a common feature. Noteworthy, accurate grading of bone marrow fibrosis has been confirmed by several groups to be prognostically informative in PMF [41, 42]. Peripheral blood leuko-erythroblastosis and anisopoikilocytosis (with tear-drop erythrocytes) correlate with the increase of bone marrow fibrosis.

Progression to AP and BP in PMF is defined by the documentation of 10–19% and 20% or more of peripheral blood or bone marrow blasts, respectively. In the bone marrow biopsy, immunohistochemistry with CD34 can facilitate the identification of increased blasts. Along these lines, identification of progenitor clusters and/or their paratrabecular localization has been shown to indicate early disease progression.

### Genetic profile

Cytogenetics abnormalities accumulate in overt-PMF and can be identified in 30–40% of patients. A number of chromosomal abnormalities have been associated with a worse outcome, in particular those defined by the Dynamic International Prognostic Scoring System-plus: complex karyotype or single or two abnormalities including 8, 7/7q-, i(17q), 5/5q-, 12p-, inv(3﻿), or 11q23 rearrangement. More recently, a three-tiered risk model has been proposed including a “very high risk (VHR)”- single/multiple abnormalities of -7, i(17q), inv(3)/3q21, 12p- /12p11.2, 11q-/11q23, or other autosomal trisomies not including +8/+9. In this cytogenetically defined group of patients, a 5-year survival rate of only 8% has been reported independent of clinically derived prognostic systems, the presence of driver and non-driver mutations, contrasting a 45% survival rate for patients with “favorable” karyotype [43]. More than 80% of patients with overt-PMF carry variants/mutations other than *JAK2/CALR/MPL*, in particular high-risk mutations which are associated with overall prognosis and leukemia-free survival (*ASXL1, SRSF*2*, IDH1/IDH2, EZH2*) [35].

Diagnostic criteria for the diagnosis of PMF according to the ICC are reported in Table 3.

**Table 3:** Diagnostic criteria for early/pre-fibrotic and overt primary myelofibrosis (PMF) according to the International Consensus Classification^1^

	Early/pre-fibrotic—PMF	Overt—PMF
Major criteria	1. Bone marrow biopsy showing megakaryocytic proliferation and atypia^a)^, bone marrow fibrosis grade < 2, increased age-adjusted BM cellularity, granulocytic proliferation, and (often) decreased erythropoiesis	1. Bone marrow biopsy showing megakaryocytic proliferation and atypia^a^, accompanied by reticulin and/or collagen fibrosis grades 2 or 3
	2. *JAK2*, *CALR*, or *MPL* mutation^b^ or presence of another clonal marker^c^ or absence of reactive bone marrow reticulin fibrosis^d^	2. *JAK2*, *CALR*, or *MPL* mutation^b^ or presence of another clonal marker^c^ or absence of reactive bone marrow reticulin fibrosis^d^
	3. Diagnostic criteria for *BCR::ABL1* positive chronic myeloid leukemia, polycythemia vera, essential thrombocythemia, myelodysplastic syndromes, or other myeloid neoplasms^e^ are not met	3. Diagnostic criteria for *BCR::ABL1* positive chronic myeloid leukemia, polycythemia vera, essential thrombocythemia, myelodysplastic syndromes, or other myeloid neoplasms^e^ are not met
Minor criteria	1. Anemia not attributed to a comorbid condition	1. Anemia not attributed to a comorbid condition
	2. Leukocytosis ≥ 11 × 10^9^/L	2. Leukocytosis ≥ 11 × 10^9^/L
	3. Palpable splenomegaly	3. Palpable splenomegaly
	4. Lactate dehydrogenase level above the reference range	4. Lactate dehydrogenase level above the reference range
		5. Leukoerythroblastosis

The diagnosis of pre-PMF or overt-PMF requires all 3 major criteria and at least 1 minor criterion confirmed in 2 consecutive determinations

^a^Morphology of megakaryocytes in pre-PMF and overt PMF usually demonstrates a higher degree of megakaryocytic atypia than in any other MPN-subtype; distinctive features of megakaryocytes include small to giant megakaryocytes with a prevalence of severe maturation defects (cloud-like, hypolobulated and hyperchromatic nuclei) and presence of abnormal large dense clusters (mostly > 6 megakaryocytes lying strictly adjacent)

^b^It is recommended to use highly sensitive assays for *JAK2* V617F (sensitivity level < 1%) and *CALR* and *MPL* (sensitivity level 1−3%)—in negative cases, consider searching for non-canonical *JAK2* and *MPL* mutations.

^c^Assessed by cytogenetics or sensitive NGS techniques; detection of mutations associated with myeloid neoplasms (e.g., *ASXL1*, *EZH2*, *IDH1*, *IDH2*, *SF3B1*, *SRSF2*, and *TET2* mutations) supports the clonal nature of the disease

^d^Minimal reticulin fibrosis (grade 1) secondary to infection, autoimmune disorder or other chronic inflammatory conditions, hairy cell leukemia or another lymphoid neoplasm, metastatic malignancy, or toxic (chronic) myelopathies

^e^Monocytosis can be present at diagnosis or develop during the course of PMF; in these cases, a history of MPN excludes CMML, whereas a higher variant allelic frequency for MPN-associated driver mutations is supporting the diagnosis of PMF with monocytosis rather than CMML

## Polycythemia vera

### Clinical features

PV incidence ranges between 0.01 and 2.8 cases per 100.00 per year. Clinically, increase of the red cell mass is mainly associated with major symptoms including hypertension, increase blood viscosity micro-circulatory symptoms, pruritus, and venous or arterial thrombosis. The latter complication is seen in about 20% of cases as the first clinical manifestation. Therefore, in the setting of splanchnic vein thrombosis and Budd-Chiari syndrome, the differential diagnosis of PV should always be considered. The cumulative risk for leukemic transformation in PV has been reported as 2.3% at 10 years and 5.5% at 15 years [44]. Risk factors for leukemic progression include advanced age, leukocytosis, abnormal karyotype, and mutations involving *SRSF2* or *IDH2*. Myelofibrotic progression consistent with post-PV MF is reported to range between 6 and 14% at 15 years [45–47].

### Morphology

**Fig. 3:**
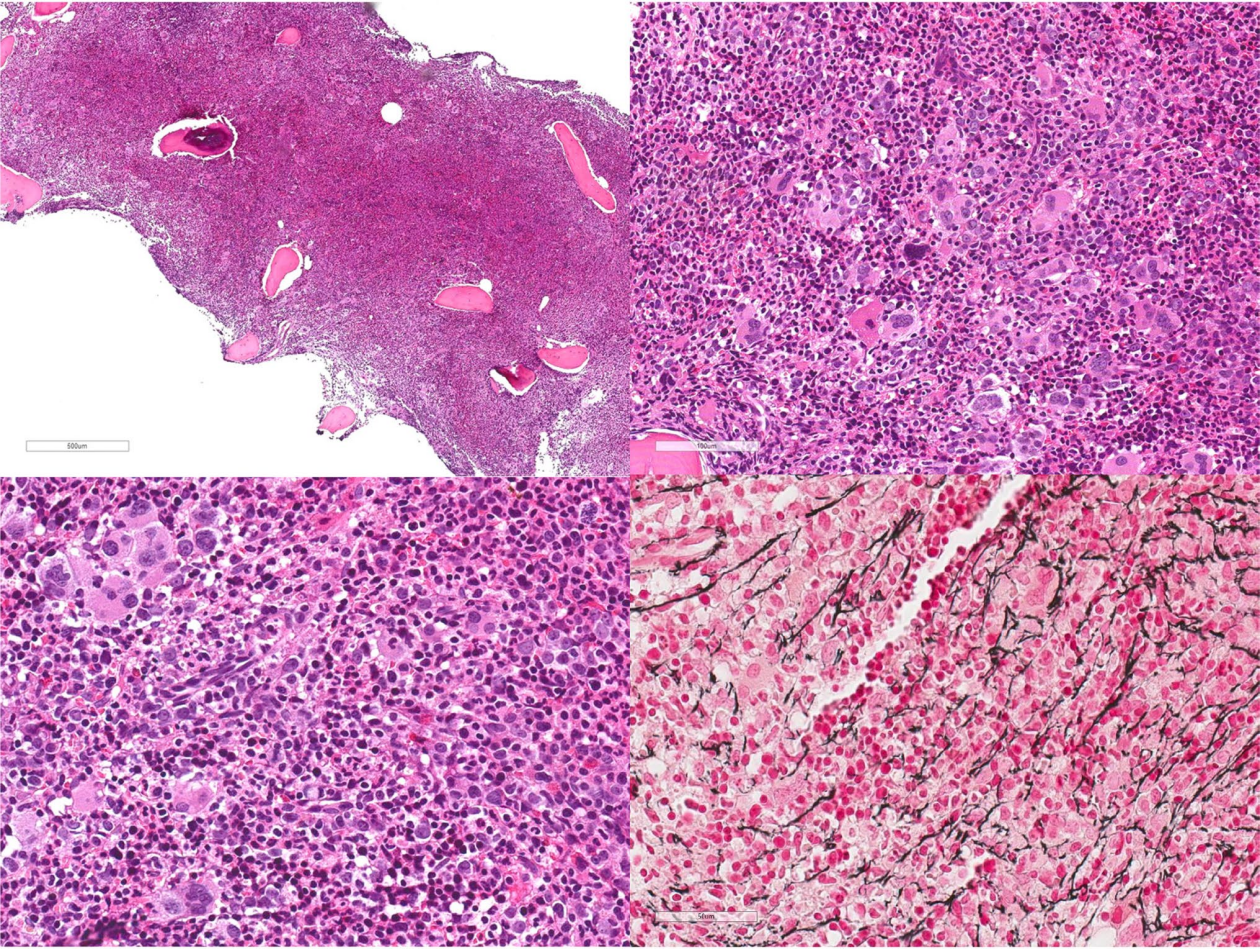
Polycythemia vera. Bone marrow is markedly hypercellular with panmyelosis. Megakaryocytes are increased in number can form loose clusters and are typically polymorphic, showing variability in size but lack significant atypia. Reticulin fibrosis can be mildly increased (MF-1)

The diagnostic thresholds for hemoglobin/hematocrit have not been changed by the ICC and therefore an acquired increase in hemoglobin/hematocrit level above 16.5 gm/dL/49% in men and 16 g/dL/48% in women, in the context of a *JAK2* mutation and characteristic bone marrow morphology define this MPN subtype. The peripheral blood shows a mild to overt excess of normochromic, normocytic RBCs. Neutrophilia and rarely basophilia may be present. The bone marrow is in almost all cases hypercellular for the patient’s age due to the proliferation of all three cell lineages (so-called panmyelosis). Erythropoiesis and granulopoiesis frequently show a left-shift, and the myeloid/erythroid ration can be variable (Fig. 3). Megakaryocytes are generally increased in number and are characterized by a marked polymorphism (marked variation in size) without any significant atypia [27, 48]. Loose clusters of megakaryocytes are a common feature in polycythemic stages of disease, whereas atypical dense and/or huge clusters as described in PMF might occur in myelofibrotic transformed end stage, i.e., post-PV MF. Noteworthy, a mild degree of reticulin fibrosis (MF-1) can be identified in about 20% of cases at initial diagnosis and has been associated with an increased risk to develop post-PV MF [49].

Diagnostic criteria for the definition of AP and BP in PV are the same as those used in ET and PMF.

### Genetic profile

*JAK2* mutational frequencies in PV are estimated at 97% for *JAK2*V617F and 3% for other activating *JAK2* mutations, including mutations in exon 12. Patients carrying *JAK2* exon 12 mutation usually present with predominant erythropoiesis, subnormal serum erythropoietin level, and younger age at diagnosis but are prognostically similar to those with *JAK2*V617F [51]. Increased allele burden does not affect survival or leukemic transformation in PV, while a higher JAK2V617F mutant allele burden might be associated with pruritus and fibrotic transformation [52]. Molecular analysis revealed in 53% of PV patients additional adverse variants (*ASXL1*, *SRSF2*, *IDH2*/*EZH2*) which correlate with inferior survival (median, 7.7 vs 16.9 years). This effect was independent of conventional prognostic models, and interestingly, the number of mutations did not provide additional prognostic information [34]. However, an abnormal karyotype has been reported in about 15–20% of patients with PV and post-PV MF and does in general contribute to a worsening of prognosis [50].

Diagnostic criteria for the diagnosis of PV according to the ICC are reported in Table 4.

**Table 4:** Diagnostic criteria for polycythemia vera (PV) and post polycythemia vera myelofibrosis (post-PV MF) according to the International Consensus Classification^1^

	PV		Post-PV MF
Major criteria	1. Elevated hemoglobin concentration or elevated hematocrit or increased red blood cell mass^a^	Required criteria	1. Previous established diagnosis of PV
	2. Bone marrow biopsy showing age-adjusted hypercellularity with trilineage proliferation (panmyelosis), including prominent erythroid, granulocytic, and increase in pleomorphic, mature megakaryocytes without atypia^b^		2. Bone marrow fibrosis of grade 2 or 3
	3. Presence of *JAK2*V617F or *JAK2* exon 12 mutation^c^		
Minor criterion	Subnormal serum erythropoietin level	Additional criteria	1. Anemia (i.e., below the reference range given age, sex, and altitude considerations) or sustained loss of requirement of either phlebotomy (in the absence of cytoreductive therapy) or cytoreductive treatment for erythrocytosis
			2. Leukoerythroblastosis
			3. Increase in palpable splenomegaly of > 5 cm from baseline or the development of a newly palpable splenomegaly
			4. Lactate dehydrogenase level above the reference range

The diagnosis of PV requires all 3 major criteria or the first two major criteria plus the minor criterion. The diagnosis of post-PV MF is established by all two required criteria and at least two additional criteria

^a^Diagnostic thresholds: hemoglobin: > 16.5 g/dL in men and > 16.0 g/dL in women; hematocrit: > 49% in men and > 48% in women; red blood cell mass: > 25% above mean normal predicted value

^b^A bone marrow biopsy may not be required in patients with sustained absolute erythrocytosis (hemoglobin concentrations of > 18.5 g/dL in men or > 16.5 g/dL in women and hematocrit values of > 55.5% in men or > 49.5% in women) and the presence of a *JAK2* V617F or *JAK2* exon 12 mutation

^c^It is recommended to use highly sensitive assays for *JAK2* V617F (sensitivity level < 1%)—in negative cases, consider searching for non-canonical or atypical *JAK2* mutations in exons 12–15

## Myeloproliferative neoplasm, unclassifiable

### Clinical features

MPN-U share clinical, morphological, and molecular features of MPN but do not fulfill the diagnostic criteria of a specific subtype. They account for about 5–10% of all MPN cases and can be subdivided in (i) early phase MPN; (ii) advanced fibrotic phase MPN; and (iii) MPN with concurrent inflammatory or neoplastic disorders obscuring the underlying MPN. The clinical presentation of MPN-U is variable: early phase MPN-U may display increased blood cell counts (thrombocytosis and/or leukocytosis and/or erythrocytosis) usually without significant splenomegaly or hepatomegaly, while advanced stages are commonly characterized by cytopenia, anemia, and organomegaly. Along these lines, about 50% of MPN patients presenting with splanchnic vein thrombosis reveal overlapping clinical and morphological features and thus are often classified as MPN-U [53].

### Morphology

In early phase MPN-U, morphological features of a specific MPN subtype are not fully developed, and many cases show overlapping features between ET and pre-PMF. Noteworthy, the reduction of the required hemoglobin/hematocrit thresholds for the diagnosis of PV by the previous WHO criteria has significantly reduced the number of unclear cases [54, 55]. If the initial diagnosis of MPN is established in the overt fibrotic phase displaying advanced stromal alterations (collagen deposition, increased micro-vessel density, sinusoid ectasia, and bone remodeling), demonstration of a characteristic driver mutation is important to establish the diagnosis, however, without assignment to a specific subtype like PMF and post-ET or post-PV MF.

### Genetic profile

Diagnosis of MPN-U can be challenging and requires the exclusion of reactive conditions, such as infections and toxin or drug exposure (growth factors, cytokines, or immunosuppressive drugs). In this context, documentation of clonality of hematopoiesis by identification of MPN driver mutations, or other mutations associated with myeloid neoplasms (*ASXL1*, *EZH2*, *TET2*, *IDH1/ IDH2*, *SRSF2*, *and SF3B1*), support the diagnosis [56]. About 20–30% of patients reveal cytogenetic abnormalities, which also support the diagnosis. Marked dysplastic changes and a lack of MPN driver mutations should prompt a careful diagnostic workup to separate these cases from MDS/MPN overlaps. In addition, it is important to highlight that a diagnosis of MPN-U cannot be made in cases with genetic lesions defining specific myeloid neoplasms (*BCR::ABL1* fusion, myeloid/lymphoid neoplasms with eosinophilia and gene rearrangement).

Diagnostic criteria for the diagnosis of CNL according to the ICC are reported in Table 5.

**Table 5:** Diagnostic criteria for Myeloproliferative neoplasms, unclassifiable (MPN-U) according to the International Consensus Classification^1^

1. Clinical and hematological features of a myeloproliferative neoplasm are present^a^	
2. *JAK2*, *CALR*, or *MPL* mutation^b^ or presence of another clonal marker^c^	
3. Diagnostic criteria for any other myeloproliferative neoplasm, myelodysplastic syndrome, myelodysplastic/myeloproliferative neoplasm^d^, or *BCR::ABL1*-positive chronic myeloid leukemia are not met	

The diagnosis of MPN-U requires all 3 criteria

^a^In cases presenting with BM fibrosis, reactive causes must be excluded, in particular BM fibrosis secondary to infection, autoimmune disorder or another chronic inflammatory condition, hairy cell leukemia or another lymphoid neoplasm, metastatic malignancy, or toxic (chronic) myelopathy

^b^It is recommended to use highly sensitive assays for *JAK2*V617F (sensitivity level < 1%) and *CALR* and *MPL* (sensitivity level 1–3%)—in negative cases, consider searching for non-canonical *JAK2* and *MPL* mutations

^c^Assessed by cytogenetics or sensitive NGS techniques; detection of mutations associated with myeloid neoplasms (e.g., *ASXL1*, *EZH2*, *IDH1*, *IDH2*, *SF3B1*, *SRSF2*, and *TET2* mutations) supports the clonal nature of the disease

^d^In cases presenting with myelodysplastic features, effects of any previous treatment, severe comorbidity, and changes during the natural progression of the disease process must be carefully excluded

## Chronic neutrophilic leukemia

### Clinical features

CNL is a rare *BCR::ABL1*-negative MPN subtype with an overall incidence of 0.1 cases/1,000,000 presenting in patients with a median age at diagnosis of 66.5 years (range: 15–86) and neutrophilic leukocytosis. In most patients, leukocytosis precedes the diagnosis for several months. Rarely patients present with symptoms, such as fatigue, bone pain, pruritus, easy bruising, or gout. Splenomegaly (and hepatomegaly) of various degree is a frequent finding and palpable splenomegaly can be detected in about 36% of *CSF3R*-mutated cases at diagnosis. Bleeding diathesis, including a high incidence of cerebral hemorrhage, can be also related to CNL [57].

### Morphology

**Fig. 4:**
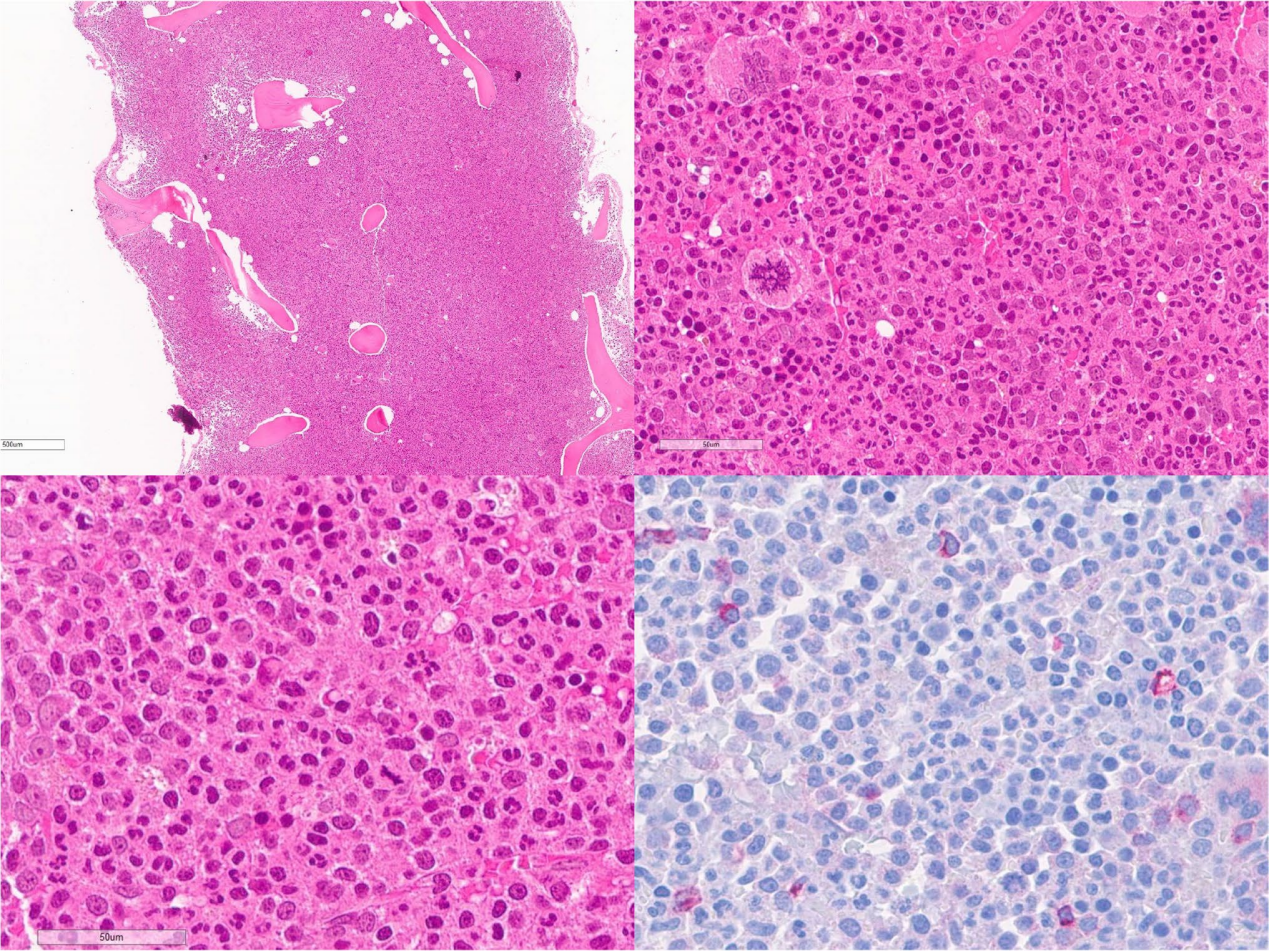
Chronic neutrophilic leukemia. Bone marrow is markedly hypercellular for the patient’s age, with hyperplastic granulopoiesis and increased number of metamyelocytes and segmented granulocytes. Megakaryocytes can be increased in number with mature morphology. Myeloblasts are usually less than 5% (CD34) (courtesy of E. Sabattini Bologna, Italy)

Bone marrow is usually markedly hypercellular (> 90% cellularity) due to the proliferating granulopoiesis with a prevalence of metamyelocytes and segmented granulocytes leading to an increased myeloid/erythroid ratio which may exceed 20:1 (Fig. 4). Erythropoiesis is usually normal, while megakaryocytes may be slightly increased, but with normal morphology. Myeloblasts usually account for less than 5% of the bone marrow cells. Monocytosis, basophilia, eosinophilia, or significant dysgranulopoiesis are usually absent, and their presence should prompt a critical review of diagnosis in order to separate the case from MDS/MPN overlaps. Mild increase in reticulin fibrosis (MF-1) can be seen in a minority of cases. In line with the other MPN subtypes, transformation to AP is defined by 10 to 19% of peripheral blood or bone marrow blasts and frequently associated with progressive splenomegaly and worsening of thrombocytopenia. Accordingly, ≥ 20% blasts define BP. In cases presenting with a *CSF3R*T618I or other activating *CSF3R* mutation, the ICC guidelines propose to lower the key diagnostic threshold for leukocytosis from ≥ 25 to ≥ 13 × 10^9^/L [58–60]. Because a marked neutrophilic increase can accompany different benign and malignant disorders, proper integration of clinical and morphological findings is mandatory for the correct differential diagnosis, in particular in molecular undefined cases. In context, the differential diagnosis includes reactive neutrophilia/leukemoid reaction, CML, and myelodysplastic/myeloproliferative neoplasms such as atypical chronic myeloid leukemia (aCML) and chronic myelomonocytic leukemia (CMML), as well as other myeloid neoplasms.

### Genetic profile

The presence of a driver mutation in the colony stimulating factor 3 receptor (*CSF3R*) is the defining genetic signature of CNL. It can be identified in 80–100% of cases, but the absence of a *CSF3R* mutation does not exclude the possibility of CNL. Among the *CSF3R*-mutated patients, two molecular subgroups (*T618I* vs other *CSF3R* mutations) with phenotypic and prognostic differences have been identified [61]. The *CSF3R*T618I-mutated subset clustered with adverse clinical and laboratory features, more advanced age at diagnosis, higher white blood cell counts, lower hemoglobin values and platelet counts at diagnosis, more frequently abnormal karyotype, and a lower overall survival in comparison to cases harboring other *CSF3R* mutations. As in other MPN subtypes, additional prognostic relevant mutations can be seen in many cases including *SETBP1*, *ASXL1*, and *SRSF2*.

Diagnostic criteria for the diagnosis of CEL, NOS according to the ICC are reported in Table 6.

**Table 6:** Diagnostic criteria for chronic neutrophilic leukemia (CNL) according to the International Consensus Classification^1^

1. Peripheral blood white blood cell count ≥ 13 × 10^9^/L^a^	
Segmented neutrophils plus banded neutrophils constitute ≥ 80% of the white blood cells	
No significant dysgranulopoiesis	
Circulating blasts only rarely observed^b^	
Monocyte count < 10% of all leukocytes	
2. Hypercellular bone marrow with neutrophil granulocytes increased in percentage and absolute number, showing normal maturation	
3. *CSF3R* T618I or another activating *CSF3R* mutation or persistent neutrophilia (≥ 3 months), splenomegaly, and no identifiable cause of reactive neutrophilia including absence of a plasma cell neoplasm or, if a plasma cell neoplasm is present, demonstration of clonality of myeloid cells by cytogenetic or molecular studies	
4. Not meeting diagnostic criteria for *BCR::ABL1*-positive chronic myeloid leukemia, polycythemia vera, essential thrombocythemia, primary myelofibrosis, or of a myeloid/lymphoid neoplasms with eosinophilia and tyrosine kinase gene fusions	

The diagnosis of CNL requires all 4 criteria

^a^≥ 25 × 10^9^/L in cases lacking *CSF3R* T618I or another activating *CSF3R* mutation

^b^10–19% blasts in peripheral blood or bone marrow represent CNL in accelerated phase (AP); > 20% blasts represent blast phase (BP)

## Chronic eosinophilic leukemia, not otherwise specified

CEL, NOS is characterized by persistent eosinophilia not meeting the criteria for other genetically defined entities. Mutations detected by NGS help to establish clonality in a significant subset of cases with eosinophilic disorders [62–64]. The bone marrow in CEL typically is hypercellular and reveals dysplastic megakaryocytes, with or without dysplastic features in other lineages, and often a significant fibrosis which is associated with the eosinophilic infiltrate. Abnormal bone marrow morphology has now been incorporated as key feature into the diagnostic criteria for CEL by the ICC guidelines in order to facilitate a better separation from related entities such as idiopathic hypereosinophilic syndrome and hypereosinophilia of unknown significance. A more detailed description of CEL, NOS, and its relationship with other myeloid neoplasms is provided in a separate article in this series “Updates on Eosinophilic Disorders.”

Diagnostic criteria for the diagnosis of CEL, NOS according to the ICC are reported in Table 7.

**Table 7:** Diagnostic criteria for chronic eosinophilic leukemia not otherwise specified (CEL, NOS) according to the International Consensus Classification^1^

1. Peripheral blood hypereosinophilia (eosinophil count ≥ 1.5 × 10^9^/L and eosinophils ≥ 10% of white blood cells)	
2. Blasts constitute < 20% cells in peripheral blood and bone marrow, not meeting other diagnostic criteria for AML^a^	
3. No tyrosine kinase gene fusion including *BCR::ABL1*, other *ABL1*, *PDGFRA*, *PDGFRB*, *FGFR1*, *JAK2*, or *FLT3* fusions	
4. Not meeting criteria for other well-defined MPN; chronic myelomonocytic leukemia, or systemic mastocytosis^b^	
5. Bone marrow shows increased cellularity with dysplastic megakaryocytes with or without dysplastic features in other lineages and often significant fibrosis, associated with an eosinophilic infiltrate or increased blasts ≥ 5% in the bone marrow and/or ≥ 2% in the peripheral blood	
6. Demonstration of a clonal cytogenetic abnormality and/or somatic mutation(s)^c^	

The diagnosis of CEL requires all 6 criteria

^a^AML with recurrent genetic abnormalities with < 20% blasts is excluded

^b^Eosinophilia can be seen in association with systemic mastocytosis (SM). However, “true” CEL, NOS may occur as SM-AMN (systemic mastocytosis with an associated myeloid malignancies)

^c^In the absence of a clonal cytogenetic abnormality and/or somatic mutation(s) or increased blasts, bone marrow findings supportive of the diagnosis will suffice in the presence of persistent eosinophilia, provided other causes of eosinophilia having been excluded

## Conclusion

In conclusion, the ICC guidelines maintain the major categories of MPN, but focus on a better definition of morphology and integration of new molecular data to improve the diagnostic definition of specific subtypes. In CML, a more simplified definition of progressive disease has been proposed, while in the other subtypes, improvement of diagnostic specificity in early disease stages has been achieved. Furthermore, highly sensitive molecular techniques for the identification of *JAK2*, *CALR*, and *MPL* driver mutations with a minimal level of VAF 1% are recommended as diagnostic backbone.
